# 616. Predicting Misdiagnoses of Infectious Disease in Emergency Department Visits

**DOI:** 10.1093/ofid/ofab466.814

**Published:** 2021-12-04

**Authors:** Alec B Chapman, Kelly Peterson, Wathsala Widanagamaachchi, Makoto M Jones

**Affiliations:** 1 University of Utah, Salt Lake City, Utah; 2 University of Washington, Salt Lake City, Utah; 3 IDEAS Center of Innovation, VA Salt Lake City Health Care System, Salt Lake City, Utah

## Abstract

**Background:**

Diagnostic error leads to delays of care and mistaken therapeutic decisions that can cascade in a downward spiral. Thus, it is important to make accurate diagnostic decisions early on in the clinical care process, such as in the emergency department (ED). Clinical data from the Electronic Health Record (EHR) could identify cases where an initial diagnosis appears unusual in context. This capability could be developed into a quality measure for feedback. To that end, we trained a multiclass machine learning classifier to predict infectious disease diagnoses following an ED visit.

**Methods:**

To train and evaluate our classifier, we sampled ED visits between December 31, 2016, and December 31, 2019, from Veterans Affairs (VA) Corporate Data Warehouse (CDW). Data elements used for prediction included lab orders and results, medication orders, radiology procedures, and vital signs. A multiclass XGBoost classifier was trained to predict one of five infectious disease classes for each ED visit based on the clinical variables extracted from CDW. Our model was trained on an enriched sample of 916,562 ED visits and evaluated on a non-enriched blind testing set of 356,549 visits. We compared our model against an ensemble of univariate Logistic Regression models as a baseline. Our model was trained to predict for an ED visit one of five infectious disease classes or “No Infection”. Labels were assigned to each ED visit based on ICD-9/10-CM diagnosis codes used elsewhere and other structured EHR data associated with a patient between 24 hours prior to an ED visit and 48 hours after.

**Results:**

Classifier performance varied across each of the five disease classes (Table 1). The classifier achieved the highest F1 and AUC for UTI, the lowest F1 for Sepsis, and the lowest AUC for URI. We compared the average precision, recall and F1 scores of the multiclass XGBoost with the ensemble of Logistic Regression models (Table 2). XGBoost achieved higher scores in all three metrics.

Table 1. Classification performance

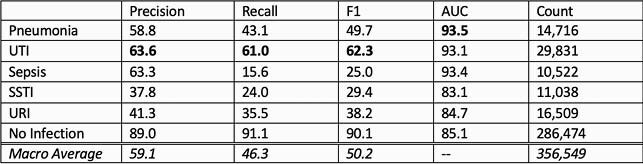

XGBoost testing set performance in each disease class, visits with no labels, and macro average. The infectious disease classes with the highest score in each metric are shown in bold.

Table 2. Baseline comparison

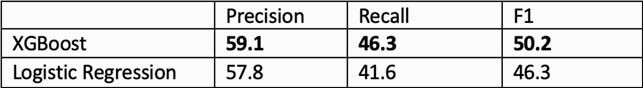

Macro average scores for XGBoost and baseline classifiers.

**Conclusion:**

We trained a model to predict infectious disease diagnoses in the Emergency Department setting. Future work will further explore this technique and combine our supervised classifier with additional signs of medical error such as increased mortality or anomalous treatment patterns in order to study medical misdiagnosis.

**Disclosures:**

**All Authors**: No reported disclosures

